# Crystal structures and hydrogen bonding in the co-crystalline adducts of 3,5-di­nitro­benzoic acid with 4-amino­salicylic acid and 2-hy­droxy-3-(1*H*-indol-3-yl)propenoic acid

**DOI:** 10.1107/S1600536814019898

**Published:** 2014-09-10

**Authors:** Graham Smith, Daniel E. Lynch

**Affiliations:** a Science and Engineering Faculty, Queensland University of Technology, GPO Box 2434, Brisbane, Queensland 4001, Australia; b Exilica Ltd, The Technocentre, Puma Way, Coventry CV1 2TT, England

**Keywords:** co-crystal structures, carb­oxy­lic acid mol­ecular adducts, 3,5-di­nitro­benzoic acid, 4-amino­salicylic acid, 2-hy­droxy-3-(1*H*-indol-3-yl)propenoic acid, hydrogen bonding, homodimers, heterodimers, π–π inter­actions.

## Abstract

The structures of the co-crystalline adducts of 3,5-di­nitro­benzoic acid (3,5-DNBA) with 4-amino­salicylic acid (PASA), the 1:1 partial hydrate, C_7_H_4_N_2_O_6_·C_7_H_7_NO_3_·0.2H_2_O, (I), and with 2-hy­droxy-3-(1*H*-indol-3-yl)propenoic acid (HIPA), the 1:1:1 *d* ^6^-dimethyl sulfoxide solvate, C_7_H_4_N_2_O_6_·C_11_H_9_NO_3_·C_2_D_6_OS, (II), are reported. The crystal substructure of (I) comprises two centrosymmetric hydrogen-bonded *R*_2_^2^(8) homodimers, one with 3,5-DNBA, the other with PASA, and an *R*_2_^2^(8) 3,5-DNBA–PASA heterodimer. In the crystal, inter-unit amine N—H⋯O and water O—H⋯O hydrogen bonds generate a three-dimensional supra­molecular structure. In (II), the asymmetric unit consists of the three constituent mol­ecules, which form an essentially planar cyclic hydrogen-bonded heterotrimer unit [graph set *R*_3_^2^(17)] through carboxyl, hy­droxy and amino groups. These units associate across a crystallographic inversion centre through the HIPA carb­oxy­lic acid group in an *R*_2_^2^(8) hydrogen-bonding association, giving a zero-dimensional structure lying parallel to (100). In both structures, π–π inter­actions are present [minimum ring-centroid separations = 3.6471 (18) Å in (I) and 3.5819 (10) Å in (II)].

## Abstract 2

The crystal structures of the co-crystal adducts of 3,5-di­nitro­benzoic acid with 4-amino­salicylic acid (a 2:2:0.4-hydrate) and with 2-hy­droxy-3-(1*H*-indol-3-yl)propenoic acid (a 1:1:1 *d* ^6^-DMSO solvate) show, respectively, polymeric and hexa­molecular hydrogen-bonded and π–π-bonded structures

## Chemical context   

3,5-Di­nitro­benzoic acid (3,5-DNBA) has been an important acid for the formation of crystalline materials, which have allowed structural characterization using single crystal X-ray methods. Most commonly proton-transfer salts are formed with organic Lewis bases, *e.g.* with 1-*H*-pyrazole (Aakeröy *et al.*, 2012 ▶) but salt–adducts are also known, *e.g.* 2-pyridyl-4′-pyrid­inium^+^–3,5-DNBA^−^–3,5-DNBA (1/1/1) (Chantra­prom­ma *et al.*, 2002 ▶). Although co-crystalline non-transfer mol­ecular adducts with 3,5-DNBA are now relatively common, inter­est was stimulated with the original reporting of non-transfer adduct formation with 4-amino­benzoic acid to form a chiral 1:1 co-crystalline material (Etter & Frankenbach, 1989 ▶), which represented one of the earliest examples of designed crystal engineering, in that case with a view to producing non-linear optical materials. In the crystalline state, carb­oxy­lic acids usually form cyclic hydrogen-bonded dimers through head-to-head carboxyl O—H⋯O hydrogen bonds (Leiserowitz, 1976 ▶) [graph set 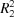
(8)]. This is the case with 3,5-DNBA (*A*), which when co-crystallized with certain aromatic acids, *e.g.* 4-(*N,N*-di­methyl­amino)­benzoic acid (*B*), gives separate mixed *AA* and *BB* homodimer pairs (Sharma *et al.*, 1993 ▶). Although uncommon with 3,5-DNBA, with other aromatic acid analogues, *AB* heterodimer formation appears more prevalent, *e.g.* the 1:1 adducts of 3,5-di­nitro­cinnamic acid with 4-(*N,N*-di­methyl­amino)­benzoic acid and 2,4-di­nitro­cinnamic acid with 2,5-di­meth­oxy­cinnamic acid (Sharma *et al.*, 1993 ▶). In both *AA* and *BB* structure types, π–π inter­actions are commonly involved in stabilization, usually accompanied by enhanced colour generation. Absence of dimer pairs in 3,5-DNBA adducts is usually the result of preferential hydrogen bonding with solvent mol­ecules, such as is found in the structure of 3,5-DNBA–phen­oxy­acetic acid–water (2/1/1) (Lynch *et al.*, 1991 ▶), in which a cyclic 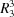
(10) inter­action is found, involving two 3,5-DNBA mol­ecules and the water mol­ecule. The title adducts C_7_H_4_N_2_O_6_·C_7_H_7_NO_3_·0.2H_2_O (I) and C_7_H_4_N_2_O_6_·C_11_H_9_NO_3_·C_2_D_6_OS (II) were prepared from the inter­action of 3,5-DNBA with 4-amino­salicylic acid (PASA) and 2-hy­droxy-3-(1*H*-indol-3-yl)propenoic acid (HIPA), respectively, and the structures are reported herein. With (II), the incorporation of C_2_D_6_OS resulted from recrystallization from *d* ^6^-di­methyl­sulfoxide.
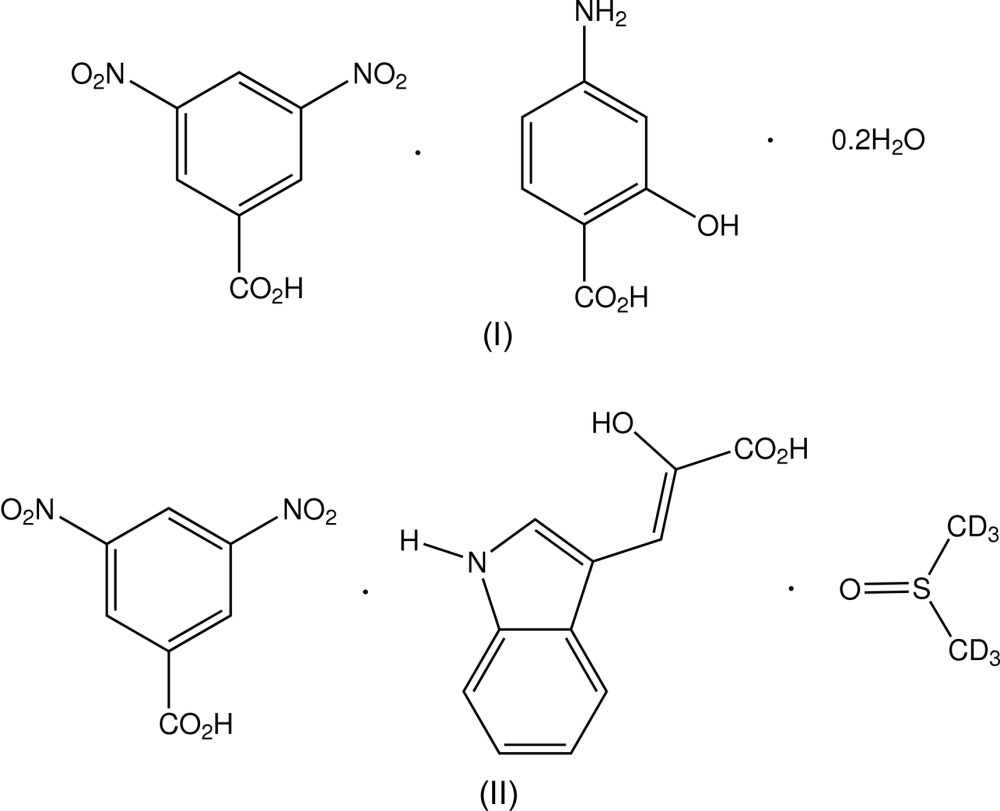


## Structural commentary   

**Figure 1:**
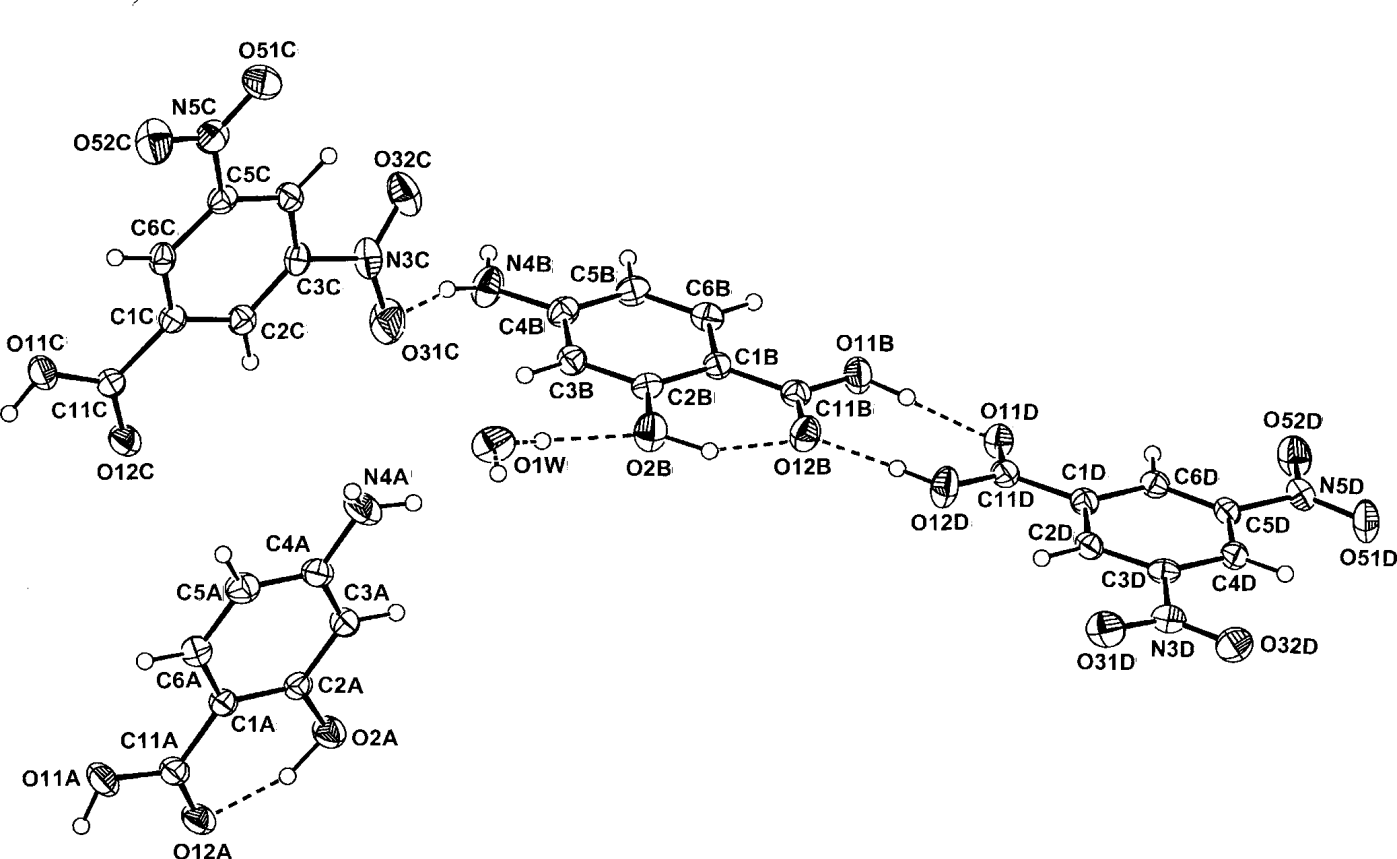
Mol­ecular conformation and atom-naming scheme for the two PABA mol­ecules (*A* and *B*), the two 3,5-DNBA mol­ecules (*C* and *D*) and the disordered water mol­ecule (O1*W*) in the asymmetric unit of adduct (I), with displacement ellipsoids drawn at the 40% probability level. Inter-species hydrogen bonds are shown as dashed lines.

In the co-crystal of 3,5-DNBA with 4-amino­salicylic acid, (I) (Fig. 1 ▶), the asymmetric unit consists of two PASA mol­ecules (*A* and *B*), two 3,5-DNBA mol­ecules (*C* and *D*) and a partially occupied water mol­ecule of solvation (O1*W*), with site occupancy = 0.4. However, what is most unusual in this structure is the presence of not four homodimers in the unit cell, but two homodimers (centrosymmetric PASA *A–A^i^* and 3,5-DNBA *C–C^ii^* pairs), as well as two heterodimer *B–D* pairs (for symmetry codes, see Table 1 ▶). All dimers are formed through the common cyclic 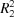
(8) ring motif. Present in the PASA mol­ecules are the expected intra­molecular salicylic acid phenolic O—H⋯O_carbox­yl_ hydrogen bonds, also present in the parent acid (Montis & Hursthouse, 2012 ▶).

**Figure 2:**
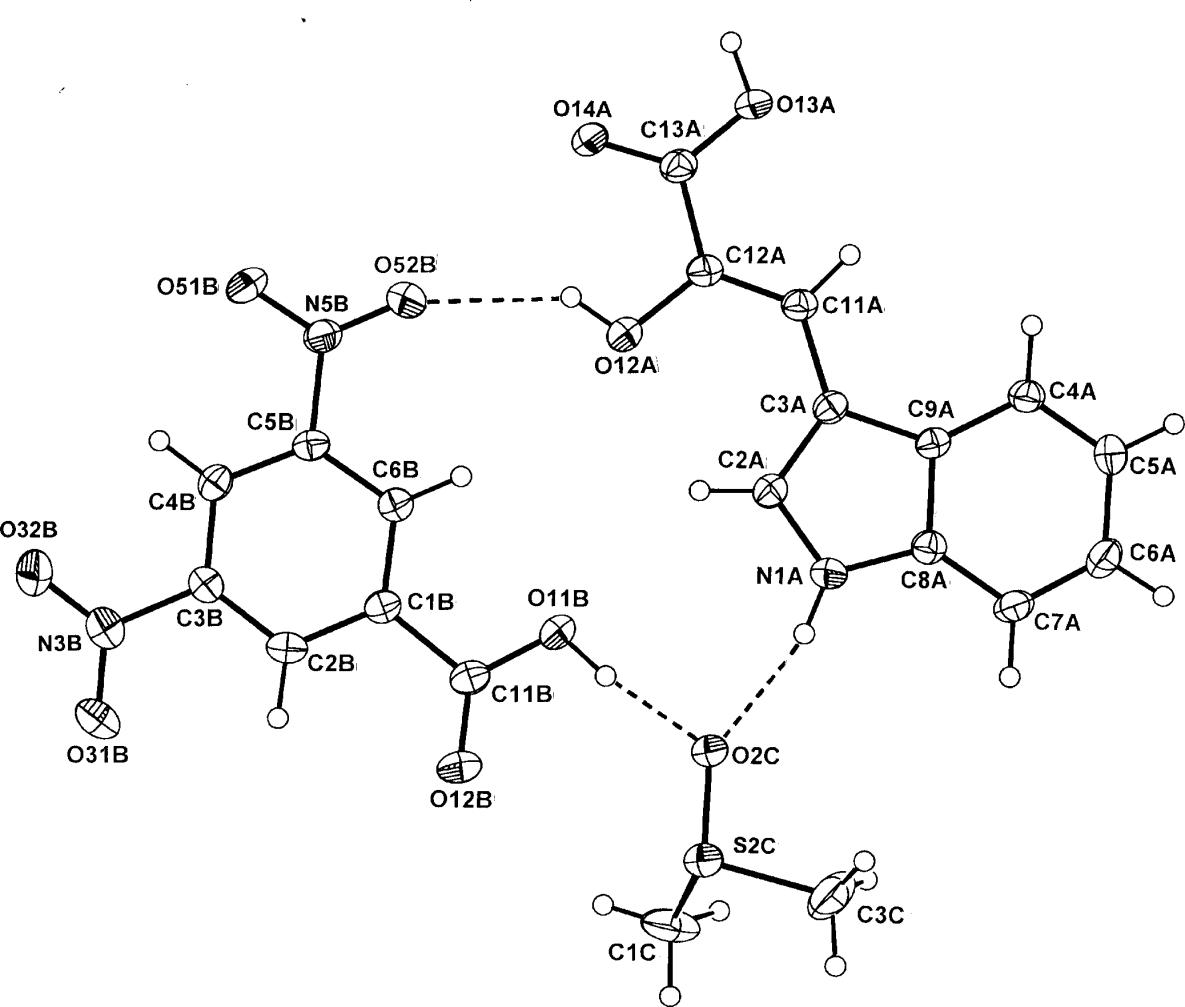
Mol­ecular conformation and atom-naming scheme for adduct (II), with displacement ellipsoids drawn at the 40% probability level. Inter-species hydrogen bonds are shown as dashed lines.

In the ternary co-crystal of 3,5-DNBA (*B*) with 2-hy­droxy-3-(1*H*-indol-3-yl)propenoic acid (*A*) and *d* ^6^-di­methyl­sulfoxide (*C*), (II) the three mol­ecules inter-associate through carb­oxy­lic acid O—H⋯O and N—H⋯O hydrogen bonds, forming a cyclic 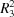
(17) heterotrimeric asymmetric unit (Fig. 2 ▶). This unit is essentially planar with a dihedral angle of 4.97 (7)° between the indole ring of *A* and the benzene ring of *B*. With the HIPA mol­ecule there is a maximum deviation from the least-squares plane of the 15-atom mol­ecule of 0.120 (2) Å (C6*A*). The planar conformation of the acid side chain in this mol­ecule is maintained by the presence of delocalization extending from C2*A* of the ring to O14*A* of the carb­oxy­lic acid group [torsion angle C11*A*—C12*A*—C13*A*—O14*A =* −177.43 (16)°]. This is also found in the parent acid, which has the similar *enol* configuration as in (II) [corresponding torsion angle 170.0 (3)°] with an *E* orientation and in the crystal forms a centrosymmetric homodimer with an 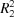
(8) hydrogen-bond motif (Okabe & Adachi, 1998 ▶).

In the adducts (I) and (II), the 3,5-DNBA mol­ecules are essentially planar with the exception of the C3-nitro groups of the *C* mol­ecule in (I), and the *B* mol­ecule in (II), where the defining C2—C3—N3—O32 torsion angles are 158.2 (3) and 168.39 (17)°, respectively. The overall torsion angle range for the remaining groups in both (I) and (II) is 170.8 (3)–179.2 (2)°. These minor deviations from planarity are consistent with conformational features of both polymorphs of the parent acid 3,5-DNBA (Prince *et al.*, 1991 ▶) and in examples both of its salts (Aakeröy *et al.*, 2012 ▶) and its adducts (Aakeröy *et al.*, 2001 ▶; Jones *et al.*, 2010 ▶; Chadwick *et al.*, 2009 ▶).

## Supra­molecular features   

**Figure 3:**
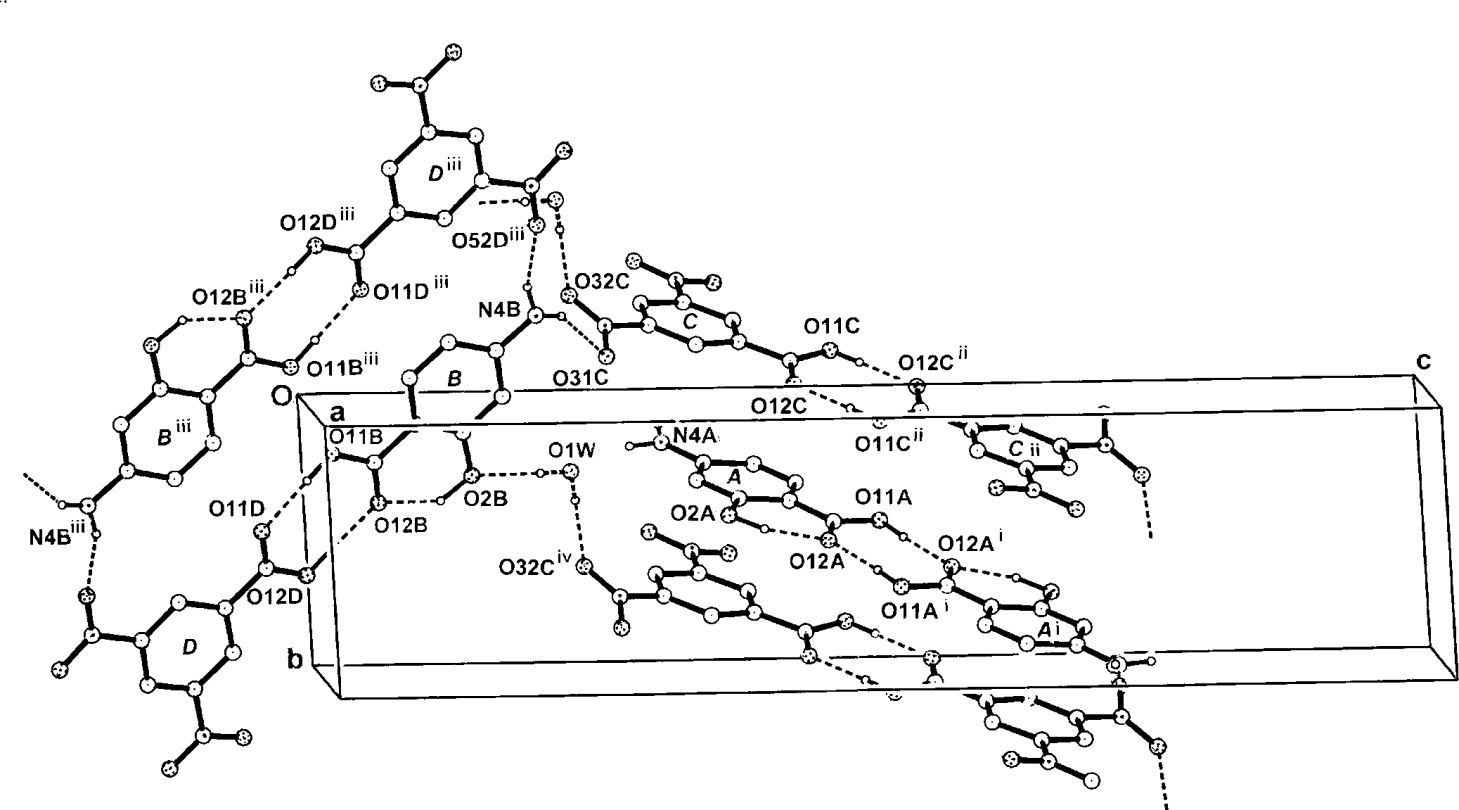
A partial expansion in the three-dimensional hydrogen-bonded structure of the adduct (I) in the unit cell, viewed down *a*. Non-associative H atoms have been omitted. For symmetry codes, see Table 1 ▶.

In the supra­molecular structure of (I), the carb­oxy­lic acid dimers are extended through inter-dimer or inter-heterodimer amine N—H⋯O and water O—H⋯O hydrogen bonds (Table 1 ▶), giving a three-dimensional framework structure (Fig. 3 ▶). Within the structure there are a number of inter-ring π–π associations [ring-centroid separations: *A⋯C* ^vi^, 3.7542 (16); *A⋯C* ^vii^, 3.6471 (16); *B⋯D* ^viii^, 3.6785 (14) Å] [symmetry codes: (vi) *x* + 1, *y* − 1, *z*; (vii) *x*, *y* − 1, *z*; (viii) −*x* + 1, −*y* + 1, −*z*]. The *B⋯D* heterodimers in the π–π association are not only related by inversion but are cyclically linked by the amine N4*B*—H⋯O52*D^iii^* hydrogen bond, forming an enlarged 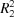
(32) ring motif. This cyclic relationship with associated π–π bonding is also found in some aromatic homodimer carb­oxy­lic acid structures (Sharma *et al.*, 1993 ▶). In (I), the disordered water mol­ecule also provides a link between the *B* mol­ecule [the phenolic O2*B* acceptor] and the *C* mol­ecule [the nitro O32*C* ^iv^ acceptor]. Also present in the structure are two very weak C—H⋯O_nitro_ inter­actions [C3*B*—H⋯O31*C* 3.382 (3) and C4*D*—H⋯O32*C* ^v^ 3.425 (3) Å; Table 1 ▶]. The H atoms of the N4*A* amine group have no acceptors with the PASA *A* homodimer unassociated in the overall structure except for the previously mentioned π–π ring inter­actions.

**Figure 4:**
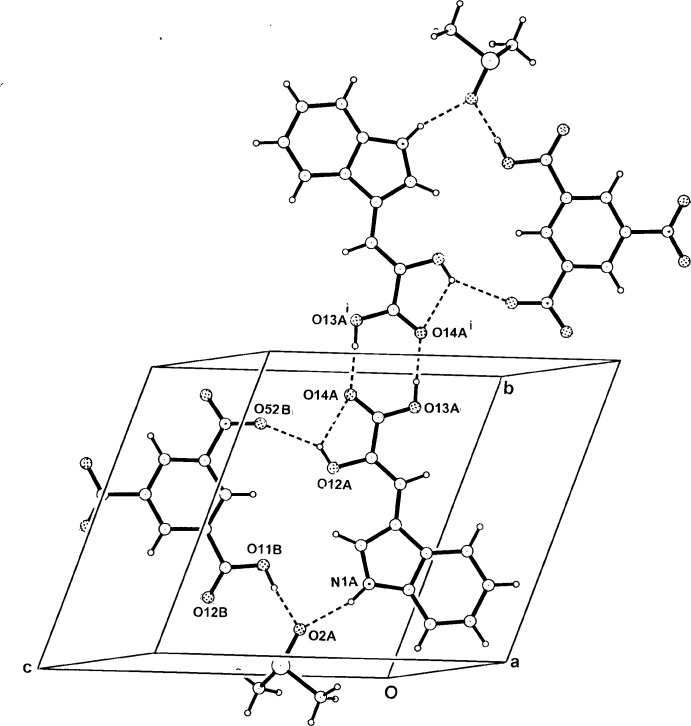
The centrosymmetric hydrogen-bonded heterohexa­meric structure of the adduct (II) in the unit cell, viewed down *a*. For symmetry code (i), see Table 2 ▶.

In (II) the hydrogen-bonded heterotrimer units associate across a crystallographic inversion centre through the HIPA carb­oxy­lic acid group [O13*A*—H⋯O14*A*]^i^ in a cyclic 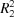
(8) hydrogen-bonding association, giving a zero-dimensional heterohexa­mer structure which is essentially planar and lies parallel to (100) (Fig. 4 ▶). Only two very weak inter­molecular *d* ^6^-DMSO methyl C—H⋯O inter­actions are present between these units inter­actions [C1*C*—D⋯O14*A* ^ii^ 3.472 (3) and C1*C*—D⋯O12*B* ^iii^ 3.372 (3) Å; Table 2 ▶]. In the structure, π–π inter­actions are also present between the benzene rings of the *A* and *B* ^viii^ mol­ecules] [minimum ring-centroid separation 3.5819 (10) Å; symmetry code: (viii) −*x*, −*y* + 2, −*z* + 1].

## Synthesis and crystallization   

The title co-crystalline adducts (I) and (II) were prepared by dissolving equimolar qu­anti­ties of 3,5-di­nitro­benzoic acid and the respective acids 4-amino­salicylic acid [for (I)] or (1*H*-indol-3-yl)propenoic acid [for (II)] in ethanol and heating under reflux for 5 min after which room-temperature evaporation of the solutions gave for (I), yellow prisms and for (II), a red powder. This latter compound was dissolved in *d* ^6^-deuterated DMSO and solvent diffusion of water into this solution gave red prisms of (II). Specimens were cleaved from both prismatic crystals for the X-ray analyses.

## Refinement details   

Crystal data, data collection and structure refinement details are summarized in Table 3 ▶. Hydrogen atoms on all potentially inter­active O—H and N—H groups in all mol­ecular species were located by difference-Fourier methods and positional and displacement parameters were refined for all but those of the phenolic O2*A* and O2*B* groups and on the disordered water molecule O1*W*, with riding restraints [O—H bond length = 0.90 (2) Å and *U*_iso_(H) = 1.5*U*_eq_(O) or N—H = 0.88 (2) Å, with *U*_iso_(H) = 1.2*U*_eq_(N)]. The phenolic and water H atoms were set invariant with *U*_iso_(H) = 1.2*U*_eq_(O). Other H atoms were included in the refinement at calculated positions [C—H (aromatic) = 0.95 or (methyl­ene) 0.99 Å] , with *U*_iso_(H) = 1.2*U*_eq_(C), using a riding-model approximation. The site-occupancy factor for the disordered water mol­ecule of solvation was determined as 0.403 (4) [for the (2:2) 3,5-DNBA:PASA pair in the asymmetric unit] and was subsequently fixed as 0.40. In the structure of (I), the relatively large maximum residual electron density (0.835 e Å^−3^) was located 0.80 Å from H6*B*.

## Supplementary Material

Crystal structure: contains datablock(s) global, I, II. DOI: 10.1107/S1600536814019898/sj5421sup1.cif

Structure factors: contains datablock(s) I. DOI: 10.1107/S1600536814019898/sj5421Isup2.hkl

Structure factors: contains datablock(s) II. DOI: 10.1107/S1600536814019898/sj5421IIsup3.hkl

Supporting information file. DOI: 10.1107/S1600536814019898/sj5421Isup4.cml

Supporting information file. DOI: 10.1107/S1600536814019898/sj5421IIsup5.cml

CCDC references: 1022646, 1022647

Additional supporting information:  crystallographic information; 3D view; checkCIF report

## Figures and Tables

**Table 1:** Hydrogen-bond geometry (Å, °) for (I)

*D*—H⋯*A*	*D*—H	H⋯*A*	*D*⋯*A*	*D*—H⋯*A*
O11*A*—H11*A*⋯O12*A* ^i^	0.91 (3)	1.78 (3)	2.678 (3)	175 (3)
O11*B*—H11*B*⋯O11*D*	0.94 (3)	1.74 (3)	2.673 (3)	175 (3)
O11*C*—H11*C*⋯O12*C* ^ii^	0.91 (3)	1.73 (3)	2.640 (3)	177 (2)
O12*D*—H12*D*⋯O12*B*	0.90 (3)	1.71 (3)	2.610 (3)	176 (2)
N4*B*—H41*B*⋯O31*C*	0.86 (3)	2.58 (3)	3.350 (4)	150 (3)
N4*B*—H42*B*⋯O52*D* ^iii^	0.85 (2)	2.44 (3)	3.210 (4)	151 (3)
O2*A*—H2*A*⋯O12*A*	0.84	1.89	2.625 (3)	145
O2*B*—H2*B*⋯O12*B*	0.84	1.85	2.587 (3)	145
O1*W*—H11*W*⋯O2*B*	0.90	2.05	2.952 (6)	179
O1*W*—H12*W*⋯O32*C* ^iv^	0.93	2.08	3.005 (5)	179
C3*B*—H3*B*⋯O31*C*	0.95	2.58	3.382 (3)	142
C4*D*—H4*D*⋯O32*C* ^v^	0.95	2.49	3.425 (3)	170

Symmetry codes: (i) 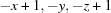; (ii) 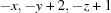; (iii) 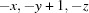; (iv) 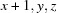; (v) 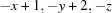.

**Table 2:** Hydrogen-bond geometry (Å, °) for (II)

*D*—H⋯*A*	*D*—H	H⋯*A*	*D*⋯*A*	*D*—H⋯*A*
N1*A*—H1*A*⋯O2*C*	0.87 (2)	2.02 (2)	2.856 (2)	161 (2)
O11*B*—H11*B*⋯O2*C*	0.88 (2)	1.72 (2)	2.591 (2)	174 (2)
O12*A*—H12*A*⋯O14*A*	0.88 (2)	2.15 (2)	2.672 (2)	118 (2)
O12*A*—H12*A*⋯O52*B*	0.88 (2)	2.20 (2)	2.951 (2)	144 (2)
O13*A*—H13*A*⋯O14*A* ^i^	0.90 (2)	1.75 (2)	2.644 (2)	178 (2)
C1*C*—D12*C*⋯O14*A* ^ii^	0.98	2.56	3.472 (3)	155
C1*C*—D13*C*⋯O12*B* ^iii^	0.98	2.52	3.372 (3)	145

Symmetry codes: (i) 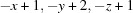; (ii) 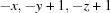; (iii) 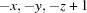.

**Table 3:** Experimental details

	(I)	(II)
Crystal data		
Chemical formula	C_7_H_4_N_2_O_6_·C_7_H_7_NO_3_·0.2H_2_O	C_7_H_4_N_2_O_6_·C_11_H_9_NO_3_·C_2_D_6_OS
*M*_r_	368.86	499.49
Crystal system, space group	Triclinic, *P* 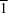	Triclinic, *P* 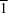
Temperature (K)	200	200
*a*, *b*, *c* (Å)	7.0717 (5), 7.5974 (4), 28.7175 (19)	7.6488 (6), 12.3552 (10), 13.3768 (10)
α, β, γ (°)	87.926 (5), 86.498 (6), 87.584 (5)	116.833 (8), 96.274 (6), 97.626 (7)
*V* (Å^3^)	1537.77 (17)	1097.40 (18)
*Z*	4	2
Radiation type	Mo *K*α	Mo *K*α
μ (mm^−1^)	0.14	0.21
Crystal size (mm)	0.35 × 0.35 × 0.30	0.45 × 0.40 × 0.32

Data collection		
Diffractometer	Oxford Diffraction Gemini-S CCD detector	Oxford Diffraction Gemini-S CCD detector
Absorption correction	Multi-scan (*CrysAlis PRO*; Agilent, 2013 ▶)	Multi-scan (*CrysAlis PRO*; Agilent, 2013 ▶)
*T*_min_, *T*_max_	0.966, 0.990	0.94, 0.98
No. of measured, independent and observed [*I* > 2σ(*I*)] reflections	10302, 6044, 4158	7457, 4310, 3490
*R*_int_	0.027	0.023
(sin θ/λ)_max_ (Å^−1^)	0.617	0.617

Refinement		
*R*[*F* ^2^ > 2σ(*F* ^2^)], *wR*(*F* ^2^), *S*	0.056, 0.149, 1.01	0.040, 0.097, 1.02
No. of reflections	6044	4310
No. of parameters	502	319
No. of restraints	8	4
H-atom treatment	H atoms treated by a mixture of independent and constrained refinement	H atoms treated by a mixture of independent and constrained refinement
Δρ_max_, Δρ_min_ (e Å^−3^)	0.86, −0.28	0.26, −0.25

Computer programs: *CrysAlis PRO* (Agilent, 2013 ▶), *SHELXS97* and *SHELXL97* (Sheldrick, 2008 ▶) within *WinGX* (Farrugia, 2012 ▶) and *PLATON* (Spek, 2009 ▶).

## supplementary crystallographic information

### Crystal data

**Table:**

C_7_H_4_N_2_O_6_·C_11_H_9_NO_3_·C_2_D_6_OS	*Z* = 2
*M**_r_* = 499.49	*F*(000) = 512
Triclinic, *P*1	*D*_x_ = 1.511 Mg m^−^^3^
Hall symbol: -P 1	Mo *K*α radiation, λ = 0.71073 Å
*a* = 7.6488 (6) Å	Cell parameters from 2343 reflections
*b* = 12.3552 (10) Å	θ = 3.3–28.4°
*c* = 13.3768 (10) Å	µ = 0.21 mm^−^^1^
α = 116.833 (8)°	*T* = 200 K
β = 96.274 (6)°	Block, red
γ = 97.626 (7)°	0.45 × 0.40 × 0.32 mm
*V* = 1097.40 (18) Å^3^	

### Data collection

**Table:**

Oxford Diffraction Gemini-S CCD detector diffractometer	4310 independent reflections
Radiation source: Enhance (Mo) X-ray source	3490 reflections with *I* > 2σ(*I*)
Graphite monochromator	*R*_int_ = 0.023
Detector resolution: 16.077 pixels mm^-1^	θ_max_ = 26.0°, θ_min_ = 3.3°
ω scans	*h* = −9→9
Absorption correction: multi-scan (*CrysAlis PRO*; Agilent, 2013)	*k* = −15→15
*T*_min_ = 0.94, *T*_max_ = 0.98	*l* = −16→16
7457 measured reflections	

### Refinement

**Table:**

Refinement on *F*^2^	Primary atom site location: structure-invariant direct methods
Least-squares matrix: full	Secondary atom site location: difference Fourier map
*R*[*F*^2^ > 2σ(*F*^2^)] = 0.040	Hydrogen site location: inferred from neighbouring sites
*wR*(*F*^2^) = 0.097	H atoms treated by a mixture of independent and constrained refinement
*S* = 1.02	*w* = 1/[σ^2^(*F*_o_^2^) + (0.0394*P*)^2^ + 0.3315*P*] where *P* = (*F*_o_^2^ + 2*F*_c_^2^)/3
4310 reflections	(Δ/σ)_max_ < 0.001
319 parameters	Δρ_max_ = 0.26 e Å^−^^3^
4 restraints	Δρ_min_ = −0.25 e Å^−^^3^

### Special details

**Table:**

Geometry. Bond distances, angles etc. have been calculated using the rounded fractional coordinates. All su's are estimated from the variances of the (full) variance-covariance matrix. The cell esds are taken into account in the estimation of distances, angles and torsion angles
Refinement. Refinement of F^2^ against ALL reflections. The weighted R-factor wR and goodness of fit S are based on F^2^, conventional R-factors R are based on F, with F set to zero for negative F^2^. The threshold expression of F^2^ > 2sigma(F^2^) is used only for calculating R-factors(gt) etc. and is not relevant to the choice of reflections for refinement. R-factors based on F^2^ are statistically about twice as large as those based on F, and R- factors based on ALL data will be even larger.

### Fractional atomic coordinates and isotropic or equivalent isotropic displacement parameters (Å^2^)

**Table:**

	*x*	*y*	*z*	*U*_iso_*/*U*_eq_	
O11B	0.11950 (19)	0.35376 (12)	0.50949 (11)	0.0386 (5)	
O12B	0.0099 (2)	0.24989 (12)	0.59702 (12)	0.0458 (5)	
O31B	−0.19578 (19)	0.48461 (14)	0.95269 (12)	0.0438 (5)	
O32B	−0.0994 (2)	0.68345 (14)	1.05107 (12)	0.0483 (5)	
O51B	0.1446 (2)	0.90405 (13)	0.86272 (12)	0.0504 (5)	
O52B	0.2503 (2)	0.80830 (13)	0.71029 (12)	0.0443 (5)	
N3B	−0.1163 (2)	0.58414 (16)	0.96484 (14)	0.0353 (6)	
N5B	0.1697 (2)	0.80906 (14)	0.78469 (13)	0.0331 (5)	
C1B	0.0493 (2)	0.46984 (16)	0.69011 (14)	0.0272 (5)	
C2B	−0.0251 (2)	0.47096 (17)	0.78086 (15)	0.0291 (6)	
C3B	−0.0359 (2)	0.58338 (17)	0.86952 (14)	0.0282 (5)	
C4B	0.0267 (2)	0.69546 (17)	0.87299 (15)	0.0289 (5)	
C5B	0.1009 (2)	0.69054 (16)	0.78195 (14)	0.0268 (5)	
C6B	0.1133 (2)	0.58021 (16)	0.68980 (15)	0.0273 (5)	
C11B	0.0569 (2)	0.34589 (17)	0.59463 (16)	0.0312 (6)	
O12A	0.36207 (19)	0.66095 (12)	0.49243 (11)	0.0372 (4)	
O13A	0.5239 (2)	0.85805 (12)	0.37886 (12)	0.0423 (5)	
O14A	0.42998 (17)	0.89974 (12)	0.54337 (11)	0.0365 (4)	
N1A	0.3546 (2)	0.28647 (14)	0.26491 (13)	0.0311 (5)	
C2A	0.3735 (2)	0.41030 (16)	0.33443 (15)	0.0293 (6)	
C3A	0.4425 (2)	0.47577 (16)	0.28182 (14)	0.0259 (5)	
C4A	0.5334 (2)	0.39011 (17)	0.08035 (15)	0.0298 (6)	
C5A	0.5337 (3)	0.28088 (18)	−0.01547 (16)	0.0369 (7)	
C6A	0.4707 (3)	0.16552 (18)	−0.02214 (17)	0.0397 (7)	
C7A	0.4090 (3)	0.15632 (17)	0.06754 (16)	0.0351 (6)	
C8A	0.4094 (2)	0.26675 (16)	0.16438 (15)	0.0280 (6)	
C9A	0.4681 (2)	0.38401 (15)	0.17227 (14)	0.0247 (5)	
C11A	0.4720 (2)	0.60721 (16)	0.32128 (15)	0.0273 (5)	
C12A	0.4337 (2)	0.69302 (16)	0.41764 (15)	0.0281 (5)	
C13A	0.4624 (2)	0.82503 (17)	0.45126 (15)	0.0309 (6)	
S2C	0.13671 (7)	0.02349 (4)	0.35794 (4)	0.0359 (2)	
O2C	0.16071 (19)	0.14384 (12)	0.35128 (11)	0.0422 (5)	
C1C	−0.0974 (3)	−0.0384 (2)	0.3210 (2)	0.0547 (9)	
C3C	0.2017 (4)	−0.0805 (2)	0.2321 (2)	0.0615 (9)	
H2B	−0.06770	0.39570	0.78180	0.0350*	
H4B	0.01890	0.77200	0.93490	0.0350*	
H6B	0.16420	0.58030	0.62820	0.0330*	
H11B	0.130 (3)	0.2799 (17)	0.4585 (17)	0.0580*	
H1A	0.308 (3)	0.2298 (16)	0.2818 (16)	0.0370*	
H2A	0.34380	0.44630	0.40820	0.0350*	
H4A	0.57650	0.46790	0.08410	0.0360*	
H5A	0.57740	0.28390	−0.07810	0.0440*	
H6A	0.47050	0.09200	−0.08990	0.0480*	
H7A	0.36810	0.07810	0.06340	0.0420*	
H11A	0.52400	0.63630	0.27430	0.0330*	
H12A	0.353 (3)	0.7300 (17)	0.5506 (16)	0.0560*	
H13A	0.540 (3)	0.9407 (15)	0.4071 (19)	0.0630*	
D11C	−0.14460	−0.04780	0.24570	0.0820*	
D12C	−0.15770	0.01790	0.37790	0.0820*	
D13C	−0.11950	−0.11940	0.31880	0.0820*	
D31C	0.13170	−0.08140	0.16570	0.0920*	
D32C	0.17950	−0.16400	0.22480	0.0920*	
D33C	0.32990	−0.05370	0.23610	0.0920*	

### Atomic displacement parameters (Å^2^)

**Table:**

	*U*^11^	*U*^22^	*U*^33^	*U*^12^	*U*^13^	*U*^23^
O11B	0.0554 (9)	0.0241 (7)	0.0344 (8)	0.0102 (6)	0.0154 (6)	0.0102 (6)
O12B	0.0661 (10)	0.0244 (8)	0.0504 (9)	0.0104 (7)	0.0179 (7)	0.0190 (7)
O31B	0.0418 (8)	0.0501 (9)	0.0519 (9)	0.0064 (7)	0.0145 (6)	0.0342 (7)
O32B	0.0582 (10)	0.0481 (10)	0.0336 (8)	0.0091 (7)	0.0151 (7)	0.0143 (7)
O51B	0.0814 (11)	0.0227 (8)	0.0397 (8)	0.0078 (7)	0.0175 (7)	0.0080 (6)
O52B	0.0587 (9)	0.0350 (8)	0.0480 (9)	0.0107 (7)	0.0234 (7)	0.0240 (7)
N3B	0.0309 (9)	0.0450 (11)	0.0361 (9)	0.0098 (7)	0.0075 (7)	0.0235 (8)
N5B	0.0404 (9)	0.0248 (9)	0.0323 (9)	0.0047 (7)	0.0051 (7)	0.0128 (7)
C1B	0.0256 (9)	0.0259 (10)	0.0301 (9)	0.0068 (7)	0.0024 (7)	0.0135 (8)
C2B	0.0256 (9)	0.0266 (10)	0.0362 (10)	0.0019 (7)	0.0006 (7)	0.0179 (8)
C3B	0.0240 (9)	0.0338 (10)	0.0274 (9)	0.0039 (7)	0.0024 (7)	0.0161 (8)
C4B	0.0288 (9)	0.0285 (10)	0.0254 (9)	0.0068 (7)	0.0013 (7)	0.0099 (7)
C5B	0.0279 (9)	0.0227 (9)	0.0292 (9)	0.0039 (7)	0.0012 (7)	0.0131 (7)
C6B	0.0266 (9)	0.0278 (10)	0.0280 (9)	0.0066 (7)	0.0036 (7)	0.0137 (7)
C11B	0.0314 (10)	0.0263 (10)	0.0352 (10)	0.0071 (7)	0.0032 (8)	0.0144 (8)
O12A	0.0537 (9)	0.0254 (7)	0.0322 (7)	0.0090 (6)	0.0146 (6)	0.0118 (6)
O13A	0.0594 (9)	0.0207 (7)	0.0456 (8)	0.0053 (6)	0.0244 (7)	0.0124 (6)
O14A	0.0424 (8)	0.0231 (7)	0.0390 (8)	0.0053 (6)	0.0160 (6)	0.0091 (6)
N1A	0.0373 (9)	0.0255 (9)	0.0350 (9)	0.0069 (7)	0.0112 (7)	0.0170 (7)
C2A	0.0317 (10)	0.0265 (10)	0.0295 (10)	0.0083 (7)	0.0075 (7)	0.0121 (8)
C3A	0.0245 (9)	0.0241 (9)	0.0271 (9)	0.0057 (7)	0.0036 (7)	0.0106 (7)
C4A	0.0287 (9)	0.0263 (10)	0.0353 (10)	0.0052 (7)	0.0091 (7)	0.0149 (8)
C5A	0.0392 (11)	0.0383 (12)	0.0359 (11)	0.0114 (9)	0.0176 (8)	0.0166 (9)
C6A	0.0452 (12)	0.0289 (11)	0.0378 (11)	0.0109 (9)	0.0148 (9)	0.0074 (8)
C7A	0.0388 (11)	0.0218 (10)	0.0410 (11)	0.0051 (8)	0.0094 (8)	0.0116 (8)
C8A	0.0263 (9)	0.0275 (10)	0.0315 (10)	0.0074 (7)	0.0062 (7)	0.0145 (8)
C9A	0.0206 (8)	0.0221 (9)	0.0302 (9)	0.0053 (7)	0.0036 (7)	0.0115 (7)
C11A	0.0249 (9)	0.0242 (9)	0.0316 (9)	0.0032 (7)	0.0051 (7)	0.0128 (7)
C12A	0.0272 (9)	0.0241 (9)	0.0312 (9)	0.0031 (7)	0.0041 (7)	0.0125 (8)
C13A	0.0265 (9)	0.0260 (10)	0.0353 (10)	0.0021 (7)	0.0064 (7)	0.0111 (8)
S2C	0.0396 (3)	0.0279 (3)	0.0398 (3)	0.0052 (2)	0.0107 (2)	0.0155 (2)
O2C	0.0567 (9)	0.0236 (7)	0.0450 (8)	0.0049 (6)	0.0255 (7)	0.0127 (6)
C1C	0.0390 (12)	0.0443 (14)	0.0970 (19)	0.0062 (10)	0.0194 (12)	0.0464 (14)
C3C	0.0669 (16)	0.0361 (14)	0.0623 (15)	0.0159 (11)	0.0213 (12)	0.0036 (11)

### Geometric parameters (Å, º)

**Table:**

S2C—C3C	1.770 (3)	C2B—H2B	0.9500
S2C—C1C	1.771 (2)	C4B—H4B	0.9500
S2C—O2C	1.5177 (17)	C6B—H6B	0.9500
O11B—C11B	1.320 (2)	C2A—C3A	1.382 (3)
O12B—C11B	1.209 (3)	C3A—C11A	1.439 (3)
O31B—N3B	1.228 (3)	C3A—C9A	1.447 (2)
O32B—N3B	1.225 (2)	C4A—C9A	1.405 (3)
O51B—N5B	1.225 (2)	C4A—C5A	1.380 (3)
O52B—N5B	1.224 (2)	C5A—C6A	1.402 (3)
O11B—H11B	0.88 (2)	C6A—C7A	1.381 (3)
O12A—C12A	1.371 (2)	C7A—C8A	1.394 (3)
O13A—C13A	1.316 (3)	C8A—C9A	1.411 (3)
O14A—C13A	1.238 (2)	C11A—C12A	1.343 (3)
O12A—H12A	0.88 (2)	C12A—C13A	1.460 (3)
O13A—H13A	0.90 (2)	C2A—H2A	0.9500
N3B—C3B	1.472 (2)	C4A—H4A	0.9500
N5B—C5B	1.472 (3)	C5A—H5A	0.9500
N1A—C8A	1.378 (2)	C6A—H6A	0.9500
N1A—C2A	1.362 (3)	C7A—H7A	0.9500
N1A—H1A	0.87 (2)	C11A—H11A	0.9500
C1B—C6B	1.388 (3)	C1C—D11C	0.9800
C1B—C2B	1.391 (2)	C1C—D12C	0.9800
C1B—C11B	1.501 (3)	C1C—D13C	0.9800
C2B—C3B	1.381 (3)	C3C—D31C	0.9800
C3B—C4B	1.382 (3)	C3C—D32C	0.9800
C4B—C5B	1.379 (2)	C3C—D33C	0.9800
C5B—C6B	1.389 (3)		
			
O2C—S2C—C1C	106.34 (11)	C4A—C5A—C6A	121.28 (19)
O2C—S2C—C3C	103.33 (11)	C5A—C6A—C7A	121.5 (2)
C1C—S2C—C3C	99.20 (13)	C6A—C7A—C8A	117.1 (2)
C11B—O11B—H11B	109.6 (15)	N1A—C8A—C9A	107.34 (16)
C12A—O12A—H12A	106.7 (15)	N1A—C8A—C7A	130.1 (2)
C13A—O13A—H13A	110.2 (14)	C7A—C8A—C9A	122.53 (17)
O32B—N3B—C3B	118.17 (19)	C4A—C9A—C8A	118.90 (17)
O31B—N3B—C3B	117.62 (17)	C3A—C9A—C8A	107.02 (16)
O31B—N3B—O32B	124.21 (18)	C3A—C9A—C4A	134.08 (19)
O51B—N5B—C5B	117.86 (16)	C3A—C11A—C12A	126.73 (18)
O52B—N5B—C5B	118.81 (16)	O12A—C12A—C11A	121.18 (19)
O51B—N5B—O52B	123.33 (19)	C11A—C12A—C13A	124.01 (18)
C2A—N1A—C8A	109.74 (17)	O12A—C12A—C13A	114.81 (15)
C2A—N1A—H1A	123.5 (13)	O14A—C13A—C12A	120.60 (18)
C8A—N1A—H1A	126.7 (13)	O13A—C13A—C12A	116.25 (16)
C2B—C1B—C6B	120.32 (17)	O13A—C13A—O14A	123.2 (2)
C6B—C1B—C11B	122.31 (16)	C3A—C2A—H2A	125.00
C2B—C1B—C11B	117.38 (19)	N1A—C2A—H2A	125.00
C1B—C2B—C3B	118.9 (2)	C5A—C4A—H4A	121.00
N3B—C3B—C4B	118.50 (17)	C9A—C4A—H4A	121.00
N3B—C3B—C2B	118.71 (19)	C4A—C5A—H5A	119.00
C2B—C3B—C4B	122.79 (17)	C6A—C5A—H5A	119.00
C3B—C4B—C5B	116.62 (18)	C7A—C6A—H6A	119.00
C4B—C5B—C6B	123.1 (2)	C5A—C6A—H6A	119.00
N5B—C5B—C6B	119.51 (16)	C6A—C7A—H7A	121.00
N5B—C5B—C4B	117.36 (17)	C8A—C7A—H7A	121.00
C1B—C6B—C5B	118.28 (17)	C3A—C11A—H11A	117.00
O11B—C11B—O12B	124.40 (19)	C12A—C11A—H11A	117.00
O12B—C11B—C1B	122.64 (17)	S2C—C1C—D11C	109.00
O11B—C11B—C1B	112.96 (19)	S2C—C1C—D12C	109.00
C1B—C2B—H2B	121.00	S2C—C1C—D13C	110.00
C3B—C2B—H2B	121.00	D11C—C1C—D12C	109.00
C3B—C4B—H4B	122.00	D11C—C1C—D13C	109.00
C5B—C4B—H4B	122.00	D12C—C1C—D13C	110.00
C5B—C6B—H6B	121.00	S2C—C3C—D31C	109.00
C1B—C6B—H6B	121.00	S2C—C3C—D32C	109.00
N1A—C2A—C3A	109.91 (16)	S2C—C3C—D33C	109.00
C2A—C3A—C11A	128.34 (16)	D31C—C3C—D32C	109.00
C9A—C3A—C11A	125.53 (17)	D31C—C3C—D33C	109.00
C2A—C3A—C9A	105.98 (17)	D32C—C3C—D33C	109.00
C5A—C4A—C9A	118.7 (2)		
			
O31B—N3B—C3B—C2B	−11.4 (2)	C4B—C5B—C6B—C1B	−0.6 (2)
O31B—N3B—C3B—C4B	169.03 (16)	N1A—C2A—C3A—C9A	−0.36 (19)
O32B—N3B—C3B—C2B	168.39 (17)	N1A—C2A—C3A—C11A	175.38 (16)
O32B—N3B—C3B—C4B	−11.2 (2)	C2A—C3A—C9A—C4A	−179.69 (18)
O51B—N5B—C5B—C4B	−5.6 (2)	C2A—C3A—C9A—C8A	0.95 (18)
O51B—N5B—C5B—C6B	174.61 (16)	C11A—C3A—C9A—C4A	4.4 (3)
O52B—N5B—C5B—C4B	173.68 (16)	C11A—C3A—C9A—C8A	−174.95 (15)
O52B—N5B—C5B—C6B	−6.2 (2)	C2A—C3A—C11A—C12A	−1.6 (3)
C2A—N1A—C8A—C9A	0.98 (19)	C9A—C3A—C11A—C12A	173.37 (17)
C8A—N1A—C2A—C3A	−0.4 (2)	C9A—C4A—C5A—C6A	−0.1 (3)
C2A—N1A—C8A—C7A	−177.76 (19)	C5A—C4A—C9A—C3A	−177.79 (19)
C6B—C1B—C2B—C3B	0.8 (2)	C5A—C4A—C9A—C8A	1.5 (2)
C2B—C1B—C11B—O11B	176.16 (15)	C4A—C5A—C6A—C7A	−1.3 (3)
C2B—C1B—C11B—O12B	−3.8 (2)	C5A—C6A—C7A—C8A	1.0 (3)
C6B—C1B—C11B—O11B	−3.9 (2)	C6A—C7A—C8A—N1A	179.11 (19)
C6B—C1B—C11B—O12B	176.13 (17)	C6A—C7A—C8A—C9A	0.5 (3)
C11B—C1B—C6B—C5B	−179.91 (15)	N1A—C8A—C9A—C3A	−1.18 (18)
C11B—C1B—C2B—C3B	−179.31 (15)	N1A—C8A—C9A—C4A	179.34 (15)
C2B—C1B—C6B—C5B	0.0 (2)	C7A—C8A—C9A—C3A	177.68 (17)
C1B—C2B—C3B—N3B	179.41 (15)	C7A—C8A—C9A—C4A	−1.8 (3)
C1B—C2B—C3B—C4B	−1.0 (3)	C3A—C11A—C12A—O12A	1.4 (3)
C2B—C3B—C4B—C5B	0.5 (2)	C3A—C11A—C12A—C13A	−178.19 (16)
N3B—C3B—C4B—C5B	−179.96 (15)	O12A—C12A—C13A—O13A	−177.22 (15)
C3B—C4B—C5B—N5B	−179.48 (15)	O12A—C12A—C13A—O14A	3.0 (2)
C3B—C4B—C5B—C6B	0.4 (2)	C11A—C12A—C13A—O13A	2.4 (2)
N5B—C5B—C6B—C1B	179.23 (15)	C11A—C12A—C13A—O14A	−177.43 (16)

### Hydrogen-bond geometry (Å, º)

**Table:**

*D*—H···*A*	*D*—H	H···*A*	*D*···*A*	*D*—H···*A*
N1*A*—H1*A*···O2*C*	0.87 (2)	2.02 (2)	2.856 (2)	161 (2)
O11*B*—H11*B*···S2*C*	0.88 (2)	2.84 (2)	3.6757 (17)	160 (2)
O11*B*—H11*B*···O2*C*	0.88 (2)	1.72 (2)	2.591 (2)	174 (2)
O12*A*—H12*A*···O14*A*	0.88 (2)	2.15 (2)	2.672 (2)	118 (2)
O12*A*—H12*A*···O52*B*	0.88 (2)	2.20 (2)	2.951 (2)	144 (2)
O13*A*—H13*A*···O14*A*^i^	0.90 (2)	1.75 (2)	2.644 (2)	178 (2)
C2*A*—H2*A*···O12*A*	0.95	2.34	2.876 (2)	115
C11*A*—H11*A*···O13*A*	0.95	2.45	2.794 (3)	101
C1*C*—D12*C*···O14*A*^ii^	0.98	2.56	3.472 (3)	155
C1*C*—D13*C*···O12*B*^iii^	0.98	2.52	3.372 (3)	145

Symmetry codes: (i) −*x*+1, −*y*+2, −*z*+1; (ii) −*x*, −*y*+1, −*z*+1; (iii) −*x*, −*y*, −*z*+1.
